# The ATR Inhibitor Elimusertib in Combination with Cisplatin in Patients with Advanced Solid Tumors: A California Cancer Consortium Phase I Trial (NCI 10404)

**DOI:** 10.1158/2767-9764.CRC-25-0305

**Published:** 2025-11-03

**Authors:** Mamta Parikh, Nataliya V. Uboha, Naoko Takebe, Kit Tam, Philippe L. Bedard, Kari B. Wisinski, Arjun Mittra, Ming Yin, Yuanquan Yang, Anne M. Noonan, Julianne L. Holleran, Christopher Ruel, Paul Frankel, Jan H. Beumer, Edward M. Newman, Alexey V. Danilov, Steven D. Gore, Primo N. Lara

**Affiliations:** 1 UC Davis Comprehensive Cancer Center, Sacramento, California.; 2 University of Wisconsin School of Medicine, Madison, Wisconsin.; 3 National Cancer Institute, Bethesda, Maryland.; 4 Princess Margaret Cancer Centre, Toronto, Canada.; 5 The Ohio State University Comprehensive Cancer Center, Columbus, Ohio.; 6 UPMC Hillman Cancer Center, Pittsburgh, Pennsylvania.; 7 City of Hope Comprehensive Cancer Center, Duarte, California.; 8 Johns Hopkins School of Medicine, Baltimore, Maryland.

## Abstract

**Purpose:**

The ataxia telangiectasia and Rad3–related kinase inhibitor elimusertib synergizes with cisplatin preclinically. We evaluated the clinical feasibility of combining elimusertib with cisplatin.

**Patients and Methods:**

Patients with advanced solid tumors who had received <300 mg/m^2^ of prior cisplatin, and for whom cisplatin-based treatment was deemed appropriate, were enrolled according to a standard 3 + 3 design, starting elimusertib at 20 mg orally twice daily on days 2 and 9, with cisplatin 60 mg/m^2^ intravenously on day 1 of a 21-day cycle. Primary objectives were the determination of the maximum tolerated dose and safety. Secondary objectives included the assessment of elimusertib pharmacokinetics and preliminary efficacy.

**Results:**

Fifteen patients were enrolled. Dose level −2 (elimusertib 20 mg once on day 2 and cisplatin 30 mg/m^2^ on days 1 and 8) was deemed the maximum tolerated dose; dose-limiting toxicities (DLT) including creatinine increase, hypokalemia, febrile neutropenia, neutropenia, syncope, and thrombocytopenia, required dose de-escalation. Although the four patients with the highest elimusertib exposure all experienced hematologic DLTs within 1 week, they also received a higher day 1 cisplatin dose, precluding a definitive association of elimusertib exposure with DLT occurrence. Of 10 evaluable patients, one (10%) with clear-cell ovarian cancer had a partial response, whereas five (50%) had stable disease.

**Conclusions:**

Cisplatin combined with elimusertib was associated with hematologic toxicity requiring significant dose de-escalation. Elimusertib pharmacokinetics was consistent with prior studies. Only modest activity was observed. Further clinical evaluation of elimusertib plus cisplatin is not warranted.

**Significance:**

Preclinical data suggest synergy between cisplatin and the ataxia telangiectasia and Rad3–related inhibitor elimusertib, leading to this phase Ib trial in advanced solid tumors evaluating feasibility. The results do not support further examination of the combination due to DLTs observed in the absence of robust efficacy.

## Introduction

Cisplatin-based chemotherapy remains an important option for many patients with advanced or metastatic malignant solid tumors. Cisplatin inhibits DNA synthesis and causes cell-cycle arrest by forming cross-links and adducts of DNA (1). However, cisplatin refractoriness or resistance develops in part due to escape from cell-cycle arrest. The ataxia telangiectasia and Rad3–related (ATR) kinase pathway is thought to be involved in this process. Furthermore, cisplatin has been shown in preclinical models to increase ATR activity transiently, lending further support to the hypothesis that ATR plays a role in cisplatin resistance (2).

Elimusertib, a highly selective and potent small-molecule ATR inhibitor, has been studied as a single agent in a trial enrolling patients with advanced solid tumors, including cohorts with colorectal cancer, castration-resistant prostate cancer, HER2-negative breast cancer, gynecologic cancers, and advanced cancers with ATM loss by IHC (3). In that study, patients were treated with elimusertib 40 mg twice daily for 3 days on and 4 days off. An alternate schedule of 3 days on and 11 days off was explored in patients whose tumors had ATM loss. Although hematologic toxicities were observed, the alternate 3 days on and 11 days off schedule was found to be better tolerated. Objective response rates were modest regardless of ATM loss or elimusertib dosing. A NCI/Cancer Therapy Evaluation Program Project Team was convened to develop early-phase clinical trial concepts for elimusertib dosing. The NCI/Cancer Therapy Evaluation Program Project Team carefully evaluated the results of completed and ongoing ATR inhibitor studies, as well as the known pharmacokinetic (PK) data for elimusertib, to establish evidence-based dose schedules for combination regimens. ATR inhibitors have demonstrated synergy when combined with cisplatin in preclinical models, including those deemed platinum-resistant (4–8). Our group also previously demonstrated synergistic effects between elimusertib and either cisplatin or carboplatin in bladder and non–small cell lung cancer cell lines (6). A modified dosing schedule seemed to be feasible based on preclinical data showing synergy between elimusertib and cisplatin. We hypothesized that we could preserve the efficacy of elimusertib with a reduced, and thus more tolerable, dosing schedule. Based on these data, we assessed the safety and tolerability of the combination of modified dosing of elimusertib with cisplatin in a phase I trial.

## Patients and Methods

### Study design

We conducted an open-label, multicenter phase Ib dose-escalation study sponsored by the NCI Experimental Therapeutics Clinical Trials Network of the combination of cisplatin and elimusertib. The elimusertib dosing schedule was based on PK and preclinical modeling indicative of activity, as well as a hypothesis that ATR activity transiently increases in response to platinum (2). Dosing escalation and de-escalation were performed using a standard 3 + 3 design. The primary objective was to evaluate the safety and tolerability and define the maximum tolerated dose (MTD) of the combination. The study was initially designed to proceed with further expansion for both this cohort and a cohort of elimusertib with gemcitabine and cisplatin, informed by the MTD, with further dose escalation/de-escalation using a 3 + 3 design. The study protocol and all amendments were approved by the NCI central Institutional Review Board as well as the appropriate individual site ethics committees. The study was performed in accordance with good clinical practice guidelines and the Declaration of Helsinki, with written informed consent obtained from all patients (clinicaltrials.gov: NCT04491942).

### Participants

Eligible patients were aged 18 years or older with histologically confirmed advanced malignant solid tumors, measurable by RECIST version 1.1 criteria and appropriate for cisplatin-based therapy. Patients were required to have adequate hematologic, renal, and hepatic function and an Eastern Cooperative Oncology Group performance status of 0 to 2. Key exclusion criteria included prior cisplatin exposure ≥300 mg/m^2^, prior treatment with any ATR inhibitor, peripheral neuropathy, or sensorineural hearing loss of grade 2 or higher by NCI Common Terminology Criteria for Adverse Events version 5.0.

### Treatment plan and assessments

**Figure 1.**
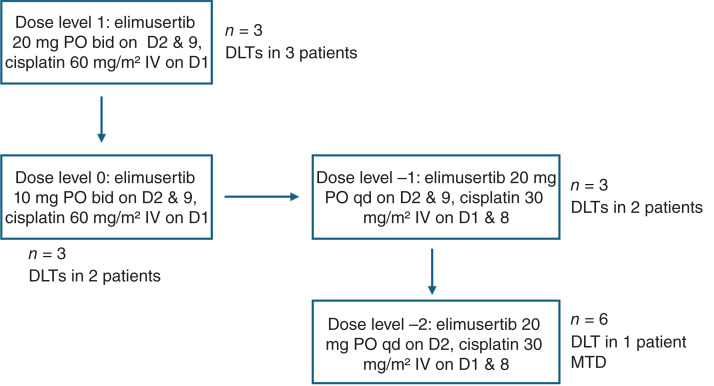
Summary of treatment de-escalation. Overview of dose de-escalation, number (*n*) of patients at each level, and number of patients experiencing a DLT at each dose level. bid, twice daily; D, day; IV, intravenously; PO, orally; qd, once a day.

Patients were treated at the first dose level (dose level 1) with cisplatin 60 mg/m^2^ intravenously on day 1 and elimusertib 20 mg twice a day orally on days 2 and 9 of a 21-day cycle for up to six cycles. Growth factor support was permitted per institutional guidelines for cisplatin. Patients were evaluable for dose-limiting toxicity (DLT) if they had received study treatment for cycle 1, including those who missed therapy due to toxicity or planned dose modifications, with nonevaluable patients replaced. Further enrollment proceeded by a 3 + 3 design; dose escalation and de-escalation were as outlined in Fig. 1. PK samples were obtained before treatment and approximately 30 minutes and 1, 1.5, 2, 4, 6, 8, and 24 hours after treatment with elimusertib, followed by PK parameter determination using noncompartmental analysis (9). DLTs were assessed during the first 21 days after starting treatment and were defined as any nonhematologic adverse event (AE) ≥ grade 3, grade 3 or higher neutropenic fever or thrombocytopenia with significant hemorrhage, grade 4 anemia, thrombocytopenia, or absolute neutrophil count. AEs were graded according to the NCI Common Terminology Criteria for Adverse Events version 5.0. Archival tumor tissue and baseline blood samples were obtained, with plans for exploratory correlative studies of whole-exome sequencing and RNA-seq to evaluate the relationship between DNA damage repair and other somatic mutations and response to therapy.

### Statistical analysis

The safety population for all safety data analyses included any patient treated with at least one dose of elimusertib or cisplatin. There was no statistical hypothesis testing in this study. Descriptive statistics were calculated for each dose level, and toxicities were tabulated and summarized.

## Results

### Patients

A total of 15 patients were enrolled in the study, with a summary of patient characteristics outlined in Table 1. Patients were evenly matched by sex, were predominantly White (*n* = 9, 60%), had a median age of 66 (range, 38–78), and were heavily pretreated with a median of three lines of prior systemic therapy (range, 1–9); the representativeness of the study population is described in Supplementary Table S2. Three patients were enrolled in dose level 1, with DLTs observed in all patients (see Table 2), and thus, three patients were enrolled at dose level 0, with two patients experiencing DLTs. After conferring with the Data Safety Monitoring Committee, the study was amended to explore additional dose levels that incorporated one-time daily dosing of elimusertib on day 2 and split dosing of cisplatin. An additional three patients were enrolled at this revised dose level −1, with two patients again experiencing DLTs. At dose level −2, three patients were enrolled, with one experiencing a DLT of grade 4 thrombocytopenia; this dose level was expanded to a total of six patients, with no further DLTs observed. Although this dose level of elimusertib 20 mg orally given once on day 2 and cisplatin 30 mg/m^2^ intravenously on days 1 and 8 was deemed the MTD, further investigation of this MTD with gemcitabine was deemed unlikely to be clinically feasible, and thus, further expansion was halted.

**Table 1.** Patient characteristics.

Patients	*N* = 15
Male (*n*, %)	7, 47%
Female (*n*, %)	8, 53%
Race/ethnicity	​
Caucasian (*n*, %)	9, 60%
Asian (*n*, %)	3, 20%
Black (*n*, %)	1, 7%
Unknown (*n*, %)	2, 13%
Age (median, range)	66, 38–78
Eastern Cooperative Oncology Group performance status	​
0 (*n*, %)	7, 47%
1 (*n*, %)	8, 53%
Primary malignancy	​
Pancreatic (*n*, %)	5, 33%
Liver/hepatocellular carcinoma (*n*, %)	2, 13%
Rectal (*n*, %)	1, 7%
Bladder (*n*, %)	2, 13%
Lung (*n*, %)	1, 7%
Breast (*n*, %)	2, 13%
Ovarian (*n*, %)	1, 7%
Cervical (*n*, %)	1, 7%
Prior radiation (*n*, %)	5, 33%
Prior lines of systemic therapy (median, range)	3, 1–9

**Table 2.** DLT summary.

Dose level (DL): regimen	Number at dose level	Patients experiencing DLTs (*n*)	DLTs observed (by patient)
DL 1: elimusertib 20 mg orally twice daily on days 2 and 9, cisplatin 60 mg/m^2^ intravenously on day 1	3	3	Patient 1 Grade 3: creatinine increase, fatigue Grade 4: neutropenia, thrombocytopenia
			Patient 2 Grade 4: neutropenia
			Patient 3 Grade 4: hypokalemia, thrombocytopenia
DL 0: elimusertib 10 mg orally twice daily on days 2 and 9, cisplatin 60 mg/m^2^ intravenously on day 1	3	2	Patient 1 Grade 4: neutropenia
			Patient 2 Grade 4: febrile neutropenia, thrombocytopenia
DL −1: elimusertib 20 mg orally once a day on days 2 and 9, cisplatin 30 mg/m^2^ intravenously on days 1 and 8	3	2	Patient 1 Grade 3: syncope
			Patient 2 Grade 4: thrombocytopenia
DL −2: elimusertib 20 mg orally once a day on day 2, cisplatin 30 mg/m^2^ intravenously on days 1 and 8	6	1	Grade 3: fatigue Grade 4: neutropenia, thrombocytopenia

### Safety

As detailed above, a summary of DLTs observed in the study is summarized in Table 2 and is notably predominant in hematologic toxicity, requiring dose de-escalation to the MTD. A detailed summary of all-grade toxicity, overall and at the MTD, is included in Supplementary Table S1. The most common (>10%) grade 3 or higher AEs were (*n*, %) lymphopenia (2, 13%), neutropenia (9, 60%), febrile neutropenia (2, 13%), leukopenia (8, 53%), anemia (5, 33%), thrombocytopenia (7, 47%), and fatigue (2, 13%). At the MTD, grade 3 or higher AEs included anemia, fatigue, leukopenia, lymphopenia, neutropenia, and thrombocytopenia.

Patients were treated with a median of two cycles (range, 1–6). Dose reductions to cisplatin were necessary in three patients due to toxicity, including two patients at the MTD level, whereas one patient at the MTD required elimusertib dose reduction due to toxicity. In dose levels that included day 9 of elimusertib, three patients out of nine required dose holds of elimusertib on day 9.

### PK

**Figure 2.**
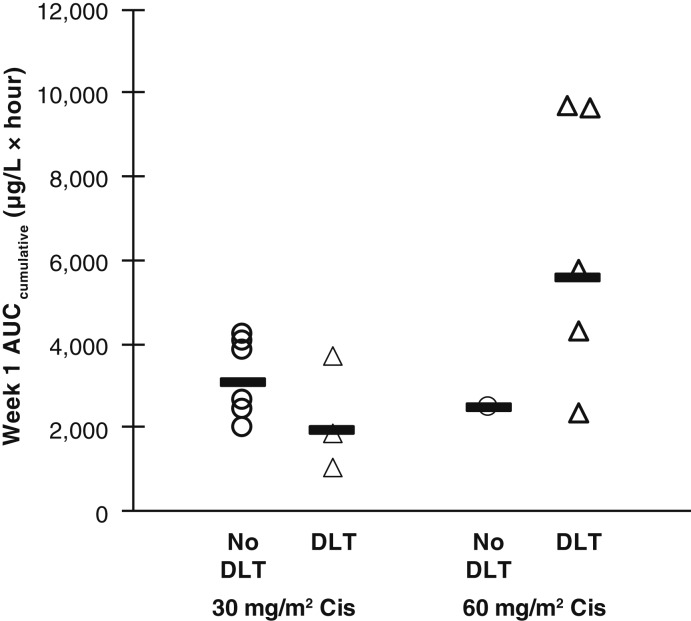
Elimusertib concentrations by toxicity. DLT occurrence and week 1 cumulative elimusertib based on day 2 AUC_0–12 hours_ by cisplatin dose. Open circles, individual patients; solid dash, geometric mean.

A summary of PK of elimusertib demonstrates that exposure is highly variable (Table 3) with a half-life of approximately 8 to 11 hours after a T_max_ of 2 to 3 hours (Supplementary Fig. S1). As most DLTs occurred during the first week, the week 1 cumulative elimusertib AUC was calculated based on AUC_0–12 hours_ (i.e., multiplied by two for the twice-daily schedule) and plotted by DLT occurrence, grouped by week 1 cisplatin dose (Fig. 2).

**Table 3.** Elimusertib geometric mean (SD in parentheses) plasma PK parameters on day 2.

Dose level	Dose (mg)	C_max_ (μg/L)	C_max_/dose (μg/L/mg)	T_max_ (hour)	t½ (hour)	AUC_0–inf_^a^ (mg/L × h)	Cl/F (L/h)	Vz/F (L)	AUC_0–12_ (mg/L × h)	AUC_0–12_/dose (μg/L × h/mg)
1	20 twice daily on days 2 and 9 (*n* = 3)	625 (1.84)	31.2 (1.84)	2.3 (2.5)	7.6 (2.0)	7.01 (1.24)	2.85 (1.24)	31.3 (1.64)	4.07 (1.35)	203 (1.35)
0	10 twice daily on days 2 and 9 (*n* = 3)	194 (1.32)	19.4 (1.32)	2.7 (2.1)	5.6 (1.9)	2.10 (1.44)	4.77 (1.44)	38.3 (1.74)	1.45 (1.40)	145 (1.40)
−1	20 once a day on days 2 and 9 (*n* = 3)	355 (2.84)	17.8 (2.84)	1.9 (3.3)	11 (1.5)	3.69 (1.74)	5.43 (1.74)	81.9 (1.71)	1.99 (1.97)	99.5 (1.97)
−2	20 once a day on day 2 (*n* = 6)	472 (1.38)	23.6 (1.38)	2.2 (2.1)	8.7 (2.0)	5.21 (1.82)	3.84 (1.82)	48.2 (1.49)	3.01 (1.36)	150 (1.36)

a For the twice-daily schedule, the last sample time was 8 hours, whereas for the once-a-day schedule, it was 24 hours. AUC_0–inf_ extrapolated beyond the last time point sampled was a geometric mean of 45% (range, 25%–77%) for the twice-daily schedule and 18% (range, 8%–45%) for the once-a-day schedule. Half-life, AUC_0–inf_, clearance, and volume of distribution are therefore best estimated in the once-a-day cohorts.

### Efficacy

At the time of data cutoff, none of the patients remained on active protocol treatment. Three patients completed study protocol therapy (*n* = 3, 20%); the other patients discontinued due to AEs (*n* = 5, 33%, including a patient who died), due to progressive disease (PD; *n* = 6, 40%), or due to physician decision (*n* = 1, 7%). Of all patients enrolled, eight patients had died at the time of data cutoff, primarily due to PD (*n* = 6, 40%). One patient died of sepsis, and another died of hemorrhagic shock; none died due to protocol therapy.

Of those evaluable for efficacy at the time of data cutoff (*n* = 10), one patient with clear-cell ovarian cancer who completed protocol-specified therapy had a confirmed partial response (PR) at the MTD. This patient was heavily pretreated, including prior platinum-based therapy and immunotherapy. She was treated at dose level −1 on the study and remained alive without additional treatment at the time of data cutoff. The remaining patients experienced either stable disease (*n* = 5, 50%) or PD (*n* = 4, 40%). In total, the objective response rate was 10%.

## Discussion

In this phase I study evaluating the combination of cisplatin and elimusertib, significant toxicity was observed at all dose levels, requiring exploration of lower dosing, less intense schedules, and dose modifications of cisplatin and elimusertib. DLTs were primarily hematologic in nature. Ultimately, the MTD dosing of cisplatin 30 mg/m^2^ intravenously on days 1 and 8 and elimusertib 20 mg once on day 2 was deemed tolerable.

The PK of elimusertib was highly variable. Observed PK parameter values for elimusertib were not dissimilar from those reported in the first-in-human study (10). T_max_ in the current study was variable and double the previously reported value of 1 hour, whereas the half-life of 8 to 11 hours was similar to the prior value of 11.5 hours. C_max_ values were very similar to the previous report, with approximately 200 μg/L for each 10 mg dose. A study conducted in parallel with the current study, combining elimusertib with FOLFIRI, similarly showed unremarkable PK that did not explain the severe toxicities (11). Because the four patients with the highest elimusertib exposure (all DLTs) in our study also received the higher day 1 cisplatin dose, the contribution of elimusertib exposure versus cisplatin dose to DLT occurrence could not be deconvoluted.

Although there were compelling preclinical data suggesting synergy between ATR inhibitors, including elimusertib, and platinum agents, this clinical trial demonstrated that further evaluation of elimusertib plus cisplatin is profoundly limited by its high toxicity. Subsequently, further plans to study the triplet of gemcitabine, cisplatin, and elimusertib, originally as part of this trial, were discontinued due to the low likelihood of establishing a tolerable therapeutic dose with the triplet.

Another ATR inhibitor, berzosertib, was previously investigated in combination with cisplatin in a phase I study of 31 patients (12). Though the most common AEs in that study were also hematologic in nature, the DLTs observed were a grade 3 hypersensitivity reaction and a grade 3 increase in alanine aminotransferase. Two patients experienced a confirmed PR despite prior platinum exposure. A subsequent randomized phase 2 study of gemcitabine and cisplatin (GC) with or without berzosertib in patients with untreated advanced urothelial carcinoma was performed by our group, showing no clinical benefit upon the addition of berzosertib (13). Hematologic toxicity rates were also higher with the combination of GC plus berzosertib, resulting in lower dose intensity of cisplatin delivered and likely inferior efficacy results when compared with the GC backbone alone.

In the current study, efficacy was modest, with only one patient with clear-cell ovarian cancer experiencing a confirmed PR. Of note, this patient was treated at a reduced dose level (dose level −1), but it is difficult to attribute efficacy to elimusertib rather than cisplatin rechallenge in a patient with ovarian cancer. In both this study and the aforementioned studies with berzosertib, ATR inhibitors were administered about 24 hours after treatment with chemotherapy, based on preclinical data suggesting that ATR is transiently upregulated after treatment with platinum agents. This did not translate clinically in terms of efficacy although, as noted, such an assessment was limited by associated toxicity. A study of elimusertib combining FOLFIRI with elimusertib pursued concomitant administration, without any appreciable improvement in efficacy and with similar toxicity concerns.

This clinical trial was limited by a lack of integrated biomarkers selecting patients more likely to respond to treatment, such as those with deficient ATM expression. Although translational correlatives are being analyzed to determine the presence of molecular alterations that might correlate to response in this study, this would be descriptive in nature and hypothesis-generating given the small sample size. Further correlative studies may be needed to identify patients more likely to benefit from elimusertib, especially in combination with other agents. Given the hematologic toxicities seen in studies combining cisplatin with berzosertib and now with elimusertib, these may represent class effects, suggesting that it may be worthwhile to consider combinations of ATR inhibitors with other agents that have a lower likelihood of overlapping toxicities.

## Supplementary Material

Supplementary Table 1 Summary of Toxicity

Supplementary Table 2 Required supplemental table of representativeness of patient population

Supplementary Figure 1 PK profiles of elimusertib

## Data Availability

Data are available upon reasonable request to the corresponding author.
