# Supplementary material for: The ATR Inhibitor Elimusertib in Combination with Cisplatin in Patients with Advanced Solid Tumors: A California Cancer Consortium Phase I Trial (NCI 10404)
Source: Cancer Res Commun. 2025 Nov 3;5(11):1946–51. doi: 10.1158/2767-9764.CRC-25-0305 (PMC12580894; doi:10.1158/2767-9764.CRC-25-0305)
Supplement: Supplementary Table 1 — Summary of Toxicity [file crc-25-0305_supplementary_table_1_suppst1.docx]

Supplemental Table 1: Summary of toxicity

|  | All patients (n=15) | |  | Patients at MTD (n=6) | |
| --- | --- | --- | --- | --- | --- |
|  | n(%) | n(%) |  | n(%) | n(%) |
| Adverse event | Grade 1-2 | Grade 3 or higher |  | Grade 1-2 | Grade 3 or higher |
| Lymphopenia^a^ | 6 (40%) | 2 (13%) |  | 3 (50%) | 1 (17%) |
| Neutropenia^a,b^ | -- | 9 (60%) |  | -- | 3 (50% |
| Febrile neutropenia^a,b^ | -- | 2 (13%) |  | -- | -- |
| Leukopenia^a,b^ | 1 (7%) | 8 (53%) |  | 2 (33%) | 2 (33%) |
| Anemia^a^ | 5 (33%) | 5 (33%) |  | -- | 3 (50%) |
| Thrombocytopenia^b^ | 1 (7%) | 7 (47%) |  | -- | 3 (50%) |
| Sepsis^c^ | -- | 1 (7%) |  | -- | -- |
| Fatigue^a^ | 7 (47%) | 2 (13%) |  | 2 (33%) | 1 (17%) |
| Anorexia | 4 (27%) | 1 (7%) |  | 2 (33%) | -- |
| Transaminitis^a^ | 1 (7%) | 1 (7%) |  | -- | -- |
| Creatinine increase^a^ | 2 (13%) | 1 (7%) |  | 1 (17%) | -- |
| Lung infection^a^ | -- | 1 (7%) |  | -- | -- |
| Syncope^a^ | -- | 1 (7%) |  | -- | -- |
| Hypokalemia^b^ | 2 (13%) | 1 (7%) |  | 2 (33%) | -- |
| Hyponatremia | 2 (13%) | -- |  | -- | -- |
| Hypomagnesemia | 5 (33%) | -- |  | 2 (33%) | -- |
| Alkaline phosphatase increase | 2 (13%) | -- |  | -- | -- |
| Dehydration | 1 (7%) | -- |  | -- | -- |
| Dyspepsia | 2 (13%) | -- |  | -- | -- |
| Epistaxis | 1 (7%) | -- |  | -- | -- |
| Gastroesophageal reflux disease | 1 (7%) | -- |  | -- | -- |
| Peripheral sensory neuropathy | 3 (20%) | -- |  | -- | -- |
| Wound dehiscence | 1 (7%) | -- |  | -- | -- |
| Diarrhea | 1 (7%) | -- |  | 1 (17%) | -- |
| Generalized muscle weakness | 1 (7%) | -- |  | -- | -- |
| Dysgeusia | 2 (13%) | -- |  | 2 (33%) | -- |
| Hypoalbuminemia | 1 (7%) | -- |  | -- | -- |

^a^Grade 3 serious adverse events (SAEs) were observed: anemia (n=4), anorexia (n=1), creatinine increase (n=1), fatigue (n=2), febrile neutropenia (n=1), leukopenia (n=4), lung infection (n=1), lymphopenia (n=2), neutropenia (n=5), transaminitis (n=1), syncope (n=1),

^b^Grade 4 SAEs were observed: febrile neutropenia (n=1), hypokalemia (n=1), leukopenia (n=4), neutropenia (n=4), thrombocytopenia (n=7)

^c^Grade 5 sepsis resulted in death in one patient
