# Supplementary material for: The ATR Inhibitor Elimusertib in Combination with Cisplatin in Patients with Advanced Solid Tumors: A California Cancer Consortium Phase I Trial (NCI 10404)
Source: Cancer Res Commun. 2025 Nov 3;5(11):1946–51. doi: 10.1158/2767-9764.CRC-25-0305 (PMC12580894; doi:10.1158/2767-9764.CRC-25-0305)
Supplement: Supplementary Table 2 — Required supplemental table of representativeness of patient population [file crc-25-0305_supplementary_table_2_suppst2.docx]

Supplementary Table 2: Representativeness of Study Participants

| Cancer types/Stage | Solid Malignant Tumors/Advanced Stage or Stage IV |
| --- | --- |
| Considerations related to: | |
| Sex | Per current cancer statistics in the United States, invasive cancer is more prevalent in men than women (about 1.1 to 1), though there are differences among different solid tumors. |
| Age | The average age of patients diagnosed with cancer is 67, according to the NCI. Again, age distribution does vary by tumor type, but our study enrolled any patient with advanced solid tumors. |
| Race/Ethnicity | Overall, Black race/ethnicity is associated with a an incidence of 0.96, American Indian/Alaska Native with incidence of 1.04, Asian American/Pacific Islander with incidence of 0.64, and Hispanic/Latino with incidence of 0.76, compared to those of White race/ethnicity. However, cancer survival after diagnosis is lower among Black patients compared to White, and this may indicate lower representation in advanced and refractory disease. |
| Geography | Our study was conducted in multiple sites geographically in the United States and Canada. In the United States, 19% of all deaths are caused by cancer, totaling a projected 618,120 cancer deaths projected to occur in the United States due to cancer in 2025. |
| Other considerations | It has been recognized for some time that in trials of cancer patients in which race/ethnicity is reported, the percentage of Black patients (quoted to be 7.3%) is well below 13.4% which is the US population average of Black individuals. Similar trends of underrepresentation are seen among Asians and Hispanic/Latino patients. Thus, this limits evaluations of the impact of racial/ethnic- or ancestry-based differences in efficacy and toxicity. |
| Overall representativeness of this study | The median age of patients in our Phase I, multi-site study was 66, which is consistent and representative of the age distribution of cancer diagnoses. In this small study of only 15 patients, we feel sex was also well-balanced, with 53% female and 47% male patients. Again, as our study was small and heterogenous in terms of tumor types, race/ethnicity representativeness was limited but consistent with many studies, with 7% Black Patients and 20% Asian, while the majority of patients (60%) were White, and 13% did not have race/ethnicity reported. |
