# Supplementary material for: The ATR Inhibitor Elimusertib in Combination with Cisplatin in Patients with Advanced Solid Tumors: A California Cancer Consortium Phase I Trial (NCI 10404)
Source: Cancer Res Commun. 2025 Nov 3;5(11):1946–51. doi: 10.1158/2767-9764.CRC-25-0305 (PMC12580894; doi:10.1158/2767-9764.CRC-25-0305)
Supplement: Supplementary Figure 1 — PK profiles of elimusertib [file crc-25-0305_supplementary_figure_1_suppsf1.docx]

Supplementary Figure 1

Supplemental Figure 1: PK profiles of elimusertib on day 2 of DL-1a (20 mg, QD, D2,9; N=6); individual patients (dashed lines, open circles) and geometric mean and SD (solid line and circles).
